# Microstructure and Fracture Mechanism Investigation of Porous Silicon Nitride–Zirconia–Graphene Composite Using Multi-Scale and In-Situ Microscopy

**DOI:** 10.3390/nano11020285

**Published:** 2021-01-22

**Authors:** Zhongquan Liao, Yvonne Standke, Jürgen Gluch, Katalin Balázsi, Onkar Pathak, Sören Höhn, Mathias Herrmann, Stephan Werner, Ján Dusza, Csaba Balázsi, Ehrenfried Zschech

**Affiliations:** 1 Fraunhofer Institute for Ceramic Technologies and Systems IKTS, Maria-Reiche-Straße 2, 01109 Dresden, Germany; Yvonne.Standke@de.bosch.com (Y.S.); juergen.gluch@ikts.fraunhofer.de (J.G.); onkar_pathak@ymail.com (O.P.); ehrenfried.zschech@ikts.fraunhofer.de (E.Z.); 2 Centre for Energy Research, Konkoly-Thege Str. 29-33, 1121 Budapest, Hungary; balazsi.katalin@ek-cer.hu (K.B.); balazsi.csaba@ek-cer.hu (C.B.); 3 Fraunhofer Institute for Ceramic Technologies and Systems IKTS, Winterbergstraße 28, 01277 Dresden, Germany; soeren.hoehn@ikts.fraunhofer.de (S.H.); mathias.herrmann@ikts.fraunhofer.de (M.H.); 4 Helmholtz Zentrum Berlin, Albert-Einstein-Straße 15, 12489 Berlin, Germany; stephan.werner@helmholtz-berlin.de; 5 Institute of Materials Research, Slovak Academy of Sciences, Watsonova 47, 040 01 Košice, Slovakia; duszaj@yahoo.com

**Keywords:** porous ceramic composite, high graphene content, GPS, multi-scale microscopy, in-situ microscopy, contact-damage resistance

## Abstract

Silicon nitride–zirconia–graphene composites with high graphene content (5 wt.% and 30 wt.%) were sintered by gas pressure sintering (GPS). The effect of the multilayer graphene (MLG) content on microstructure and fracture mechanism is investigated by multi-scale and in-situ microscopy. Multi-scale microscopy confirms that the phases disperse evenly in the microstructure without obvious agglomeration. The MLG flakes well dispersed between ceramic matrix grains slow down the phase transformation from α to β-Si_3_N_4_, subsequent needle-like growth of β-Si_3_N_4_ rods and the densification due to the reduction in sintering additives particularly in the case with 30 wt.% MLG. The size distribution of Si_3_N_4_ phase shifts towards a larger size range with the increase in graphene content from 5 to 30 wt.%, while a higher graphene content (30 wt.%) hinders the growth of the ZrO_2_ phase. The composite with 30 wt.% MLG has a porosity of 47%, the one with 5 wt.% exhibits a porosity of approximately 30%. Both Si_3_N_4_/MLG composites show potential resistance to contact or indentation damage. Crack initiation and propagation, densification of the porous microstructure, and shift of ceramic phases are observed using in-situ transmission electron microscopy. The crack propagates through the ceramic/MLG interface and through both the ceramic and the non-ceramic components in the composite with low graphene content. However, the crack prefers to bypass ceramic phases in the composite with 30 wt.% MLG.

## 1. Introduction

Materials for high temperature applications usually require tailored mechanical properties (e.g., fracture toughness, bending strength), good resistance to thermal shock, creep resistance, high thermal conductivity, as well as good tribological and wear properties [1,2,3,4]. Ceramic materials have been extensively investigated in the previous decades due to their high temperature performance in general. Silicon nitride (Si_3_N_4_) ceramics have the potential to meet the requirements mentioned above (e.g., low coefficient of thermal expansion (CTE), good thermal conductivity and high strength, resulting in a higher thermal shock resistance than most other ceramic materials), therefore, it is commonly used in a variety of structural applications such as cutting tools, pump seal parts, bearing balls, gas turbine engine parts or heat exchangers [5,6,7]. To further extend its application field, forming multi-component materials or composites is under development to tailor both the mechanical and functional properties of Si_3_N_4_ ceramics. Typical metal oxides (e.g., MgO, Al_2_O_3_, Y_2_O_3_, or ZrO_2_) in Si_3_N_4_ promote a liquid phase formation, which facilitates the consolidation [4]. The use of tetragonal zirconia as an energy-dissipation component can effectively result in an improvement of fracture toughness of Si_3_N_4_ ceramics [8,9,10]. Energy dissipation and consequently high fracture toughness of zirconia-containing ceramics can be related to the tetragonal-to-monoclinic phase transformation [9,10]. In another aspect, composites with nanofillers (e.g., carbon nanotube, graphene and hexagonal boron nitride (h-BN) show the potential to improve the properties of Si_3_N_4_-based ceramic matrix composites [11,12,13]. Due to the unique combination of electrical, thermal and mechanical properties [14], graphene and graphene oxide (GO) have been considered as components in ceramic matrix composites for the last decade. Several studies were reported on graphene reinforced Si_3_N_4_ matrix composites [15,16,17,18,19,20,21,22]. However, the problem of these composites is that graphene or graphite reacts with the sintering additives. This effect strongly reduces the densification of the material. Dense materials were observed only by hot pressing (HP) or spark plasma sintering (SPS), due to the fast densification caused by the high uniaxial pressure. Adding 1 wt.% graphene platelet (GPL) into Si_3_N_4_ with hot isostatic pressing (HIP) and gas pressure sintering (GPS) improved the fracture toughness, based on toughening mechanisms such as crack deflection, crack branching and crack bridging [15,17,18]. Seiner et al. [19] investigated the elastic constants of silicon composites with variable content (3–18 wt.%) of graphene fillers (nanoplatelets and reduced GO sheets) by resonant ultrasound spectroscopy. Young’s modulus (*E*) and shear modulus (*G*) monotonically decrease with the filler concentration for both types of fillers. Hvizdos et al. [20] and Balko et al. [21] observed no decrease in the coefficient of friction (COF) at room temperature for Si_3_N_4_ matrix nanocomposites with 3 wt.% graphene. The authors mentioned that the graphene addition resulted in an insufficient densification and pores in the composite. The presence of porosity could be a reason for no change in the COF. Rutkowski et al. [22] investigated the thermal stability and conductivity of hot-pressed Si_3_N_4_ composite with a graphene content up to 10 wt.%. Anisotropic behavior was observed with excellent thermal properties in the major directions of graphene. Although using either ZrO_2_ or graphene nanofillers to improve the properties of Si_3_N_4_ has been reported, research on the combined use of these materials is rather rare [23,24]. On the other hand, it seems that the study on the effect of further increase in graphene content up to more than 20 wt.% into Si_3_N_4_ composite remains a gap to be filled. It is also important to explore the possibility of including very high graphene content to further develop novel Si_3_N_4_ matrix composites, for instance, porous ceramic composites. Porous carbon/silicon nitride composites showed tunable and weakly negative permittivity, which is necessary for the applications in solar energy harvesting, sensor and antennas [25]. Wave-transparent porous silicon nitride was produced using gel-casting and pressureless sintering [26]. Several studies demonstrated the potential of porous ceramics/ceramic composites to sustain mechanical damage and absorb energy, which benefits their applications in energy production, filtration, water treatment, absorption and catalysis as support or coating materials [27,28,29,30,31,32].

In this study, a porous ceramic composite with high graphene content (5 wt.% and 30 wt.%) was sintered by GPS from attrition milled Si_3_N_4_ ceramics and multilayer graphene (MLG), with in-situ incorporated ZrO_2_ particles. The studied porous silicon nitride–zirconia–graphene composites show potential resistance to contact or indentation damage. Combined use of ZiO_2_ and high content of graphene results in a complex ceramic system with high porosity, which is challenging to investigate using a conventional approach in ceramic studies. Therefore, the effect of the MLG content on the microstructure and the fracture mechanism of Si_3_N_4_-ZrO_2_/graphene composites is investigated using multi-scale and in-situ microscopy. Multi-scale and in-situ microscopy as a combined methodology reported in this study also provides a potential unique approach to understand the microstructure and mechanical behavior correlation for other complex ceramic systems.

## 2. Materials and Methods

### 2.1. Silicon Nitride–Zirconia–Graphene Composite Preparation

A commercial alpha silicon nitride powder (UBE Corp., Ube, Japan, particle size: 0.6 µm, specific surface area: 4.8 m^2^/g) was used as matrix material. The base powder consisted of 90 wt.% α-Si_3_N_4_, 4 wt.% Al_2_O_3_ (Alcoa, A16, Pittsburgh, PA, USA) and 6 wt.% Y_2_O_3_ (H.C. Starck, grade C, Goslar, Germany). It was mixed by attrition milling (Union Process, type 01-HD/HDDM, Akron, OH, USA) equipped with zirconia agitator discs and ZrO_2_ grinding media (3 vol% Y_2_O_3_ stabilized, diameter of 1 mm) in a 750 cm^3^ zirconia tank. The milling process was performed at a high rotation speed of 3000 min^−1^ for 5 h in ethanol [33]. ZrO_2_ particles were incorporated into the Si_3_N_4_ during the milling, from the abrasion of zirconia balls under controlled conditions. The contribution of ZrO_2_ was adjusted between 30 and 42 wt.%. Commercial graphite powder (Aldrich, St. Louis, MO, USA, grain size: 1 µm) was milled intensively in ethanol for 10 h using the same attrition milling system, and subsequently added into powder mixture. The final powder mixture (with 5 wt.% and 30 wt.% MLG) was dried and sieved with a filter with a mesh size of 150 µm. Polyethylene glycol (PEG, 10 wt.%) surfactant and deionized water were added to the powder mixture before sintering. Samples with the dimension 5 mm × 5 mm × 50 mm were pressed by dry pressing at 220 MPa. The GPS was applied to form the final composites in nitrogen atmosphere at 1700 °C and 20 MPa for 3 h.

### 2.2. Multi-Scale and In-Situ Microscopy

High surface quality of composites was achieved by grinding, standard polishing, and ion polishing before performing scanning electron microscopy (SEM) studies. SEM imaging was performed at an operating voltage of 3 kV using a Carl Zeiss NVision 40 tool (Oberkochen, Germany), applying an Energy selective Backscattered (EsB) detector. The sample for X-ray microscopy (XRM) was firstly grinded with a relative flat surface, then a square pillar was prepared with a length of about 3 µm using focused ion beam (FIB) milling. An XRM study was carried out at the U41-XM beamline of the electron storage ring BESSY II, Helmholtz–Zentrum Berlin (Berlin, Germany). The used photon energy was 800 eV. The sample was tilted from −65° to +65° with 1° steps. The tomography was reconstructed by Tomo3D [34] (Almeria and Madrid, Spain), and rendered and sliced using the Tomviz software [35] (Ithaca, NY, USA). Standard lift-out lamellae with a thickness of about 200 nm for transmission electron microscopy (TEM) study were prepared using FIB milling, after local carbon and Pt deposition. TEM (Carl Zeiss Libra 200 Cs, Oberkochen, Germany, with an acceleration voltage of 200 kV) was used to study of the microstructure of the sintered composites. Energy-dispersive X-ray spectroscopy (EDX) was performed on the samples using a detector of Oxford Instruments attached to the TEM. A quantitative analysis of the microstructure from SEM and TEM images was performed in Fiji [36]. SEM images with magnification of 5000× and 10,000×, and TEM images with magnification of 20,000× and 30,000× were used. Si_3_N_4_ was treated as rod shape with round cross section, and ZrO_2_ was treated as globular shape in the analysis. Si_3_N_4_ phases with high aspect ratio larger than 3 were used to calculate the length and diameter of the cross section, while the rest was only used to calculate the diameter of the cross-section. The porosity was measured both by water intrusion porosimetry [21] and mercury intrusion porosimetry [37]. The density was measured applying the Archimedes method. Vickers hardness measurement (hardness tester LECO 700AT, St. Joseph, MI, USA) was performed at loads from 9.81 to 150 N, the dwelling time was 10 s in all cases. The sample for in-situ TEM experiment was H-bar sample with a thickness of about 800 nm prepared by FIB milling. A wedge indenter with a piezo control in a TEM holder was used to perform the in-situ test.

## 3. Results and Discussion

**Figure 1:**
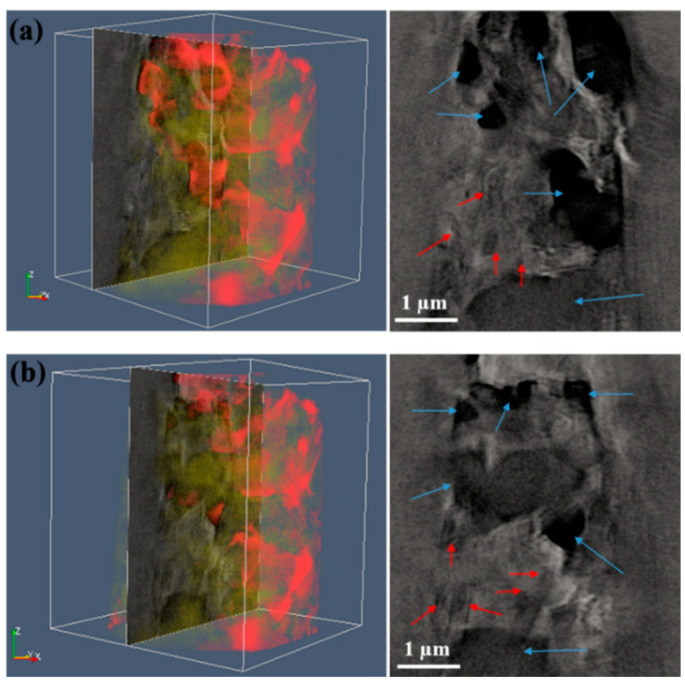
(**a**,**b**) Extracted slices from a volumetric reconstruction of the composite with 30 wt.% multilayer graphene (MLG) by synchrotron-based nano-X-ray computed tomography (XCT). ZrO_2_ phases are indicated by blue arrows, Si_3_N_4_ phases are indicated by red arrows.

A square pillar from the synthesized composite with 30 wt.% MLG was prepared for 3D microstructure studies using XRM. X-ray computed tomography (XCT) data are shown in Video S1 (supplementary materials), two extracted slices are shown in Figure 1. The ZrO_2_ phases were easily differentiated by the contrast, while only partial Si_3_N_4_ phases were distinguished from the mixed Si_3_N_4_ phase and MLG flakes. The size of major ZrO_2_ phases is less than 1 µm, the size of Si_3_N_4_ phases is less than 0.5 µm. The size of MLG flakes could not be unambiguously determined by XRM. Empty space (open pores) is clearly observed as well from the 3D tomography data as shown in Video S1 (Supplementary Materials).

**Figure 2:**
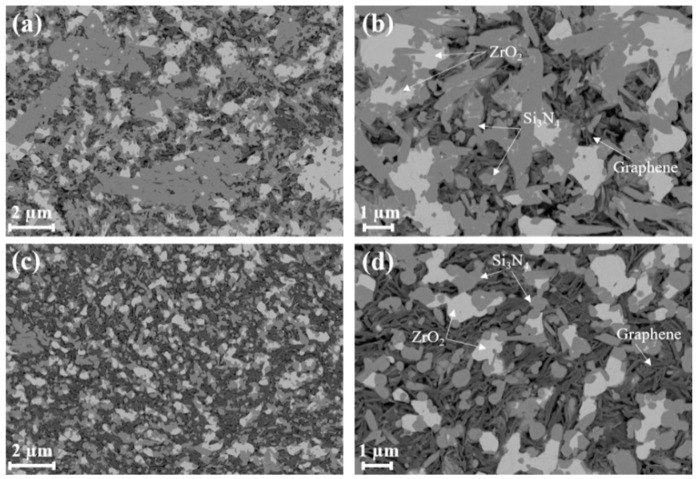
SEM images of the sintered silicon nitride–zirconia–graphene composite. (**a**,**b**) With 5 wt.% MLG; (**c**,**d**) with 30 wt.% MLG.

**Figure 3:**
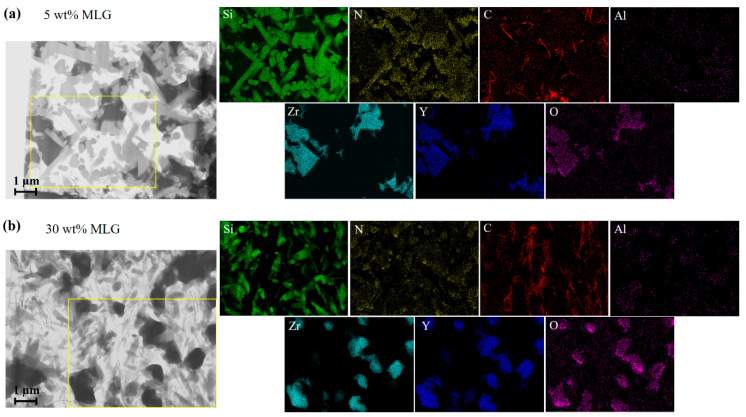
TEM image and corresponding elemental maps of the sintered composites. (**a**) With 5 wt.%, (**b**) with 30 wt.%.

**Figure 4:**
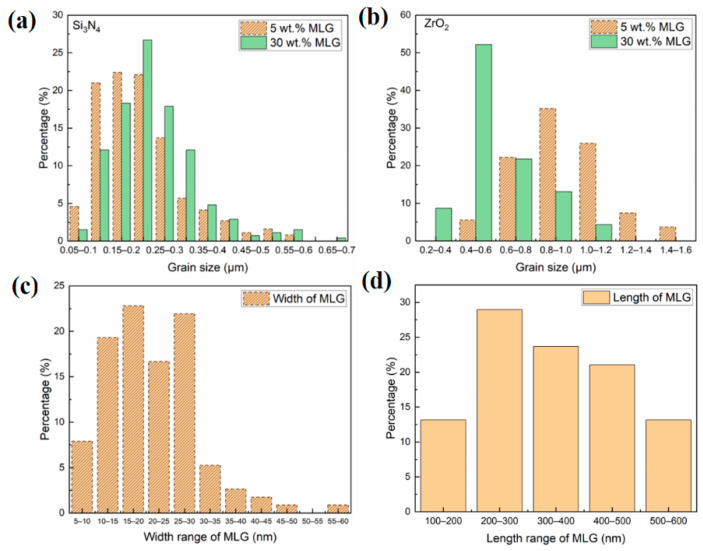
Histograms of size distribution for Si_3_N_4_ (**a**), ZrO_2_ (**b**), and MLG (**c**,**d**) in the sintered composites with different graphene content, analyzed from SEM and TEM images.

The SEM images on the grinded and ion-polished samples detected using an EsB detector show clear compositional contrast, Si_3_N_4_, ZrO_2_ and MLG flakes are easily distinguished (indicated by arrows in Figure 2). After the GPS process, spheroid ZrO_2_ particles (mostly c-ZrO_2_, as indicated by the XRD data in Figures S1 and S2) and thin MLG platelets were successfully incorporated into the Si_3_N_4_ matrix for both composites. MLG platelets were embedded and entangled among Si_3_N_4_ and ZrO_2_ phases. Slight agglomeration of ceramic particles was also observed. Hexagonal β-Si_3_N_4_ phases (rod-like) were commonly observed in the sintered composite with 5 wt.% MLG, while approximately 2.5 wt.% of α-Si_3_N_4_ phases apart from the major β-Si_3_N_4_ phases still remained in the sintered composite with 30 wt.% MLG (XRD data in Figures S1 and S2). Rod-like β-Si_3_N_4_ phases with a high aspect ratio represent the majority in the microstructure in the sample with 5 wt.% MLG addition, while β-Si_3_N_4_ phases with a low aspect ratio are more common in the sample with 30 wt.% MLG addition (Figure 2 and Figure 3). Due to the high porosity, gas phase reactions will also influence the grain growth. In the α-β phase transformation, the liquid phase is crucial in the whole process of the dissolution of the fine α-phase starting powder and subsequent precipitation of the β-phase [2]. High content addition of MLG could react with the liquid phase, in particular by the reduction in sintering additives [38,39,40]. Therefore, it slowed down both the densification and the phase transformation from α to β-Si_3_N_4_. The high content of MLG plates slowed down the subsequent needle-like growth of β-Si_3_N_4_ rod as well (Figure 2) by serving as barrier layer. The Si_3_N_4_ and ZrO_2_ phases were quantitatively analyzed using the SEM images, the results are summarized in Figure 4a,b. Compared with the sample with 5 wt.% MLG addition, the size distribution of Si_3_N_4_ phase shifts towards a larger size range for the sample with 30 wt.% MLG addition. The size (diameter) mainly ranges from 100 to 300 nm with 5 wt.% MLG addition and from 100 to 350 nm with 30 wt.% MLG addition, respectively. On the contrary, a higher MLG content (30 wt.% MLG) hinders the growth of ZrO_2_ phase (Figure 4b), in which the high content of graphene acts as barrier layer. For 5 wt.% MLG addition, the average diameter and length of hexagonal Si_3_N_4_ phases were 221 ± 9 nm and 1496 ± 39 nm, and the average size of spheroid ZrO_2_ particles was 867 ± 27 nm. For the sample with 30 wt.% MLG addition, the average diameter and length of hexagonal Si_3_N_4_ phases was 249 ± 9 nm and 1363 ± 31 nm, respectively, and the average size of the spheroid ZrO_2_ phases was 601 ± 19 nm. The average size of Si_3_N_4_ phases observed in this study is smaller than in typical monolithic Si_3_N_4_ ceramics sintered at similar conditions [17] because of the addition of MLG resulting in the reduction in the liquid phase and a high residual porosity. The volume ratio of Si_3_N_4_ phase to ZrO_2_ phase observed by image analysis was about 2.65:1 in the sample with 5 wt.% MLG, and about 2:1 in the sample with 30 wt.% MLG. Although the addition of ZrO_2_ into the initial Si_3_N_4_ powder can noticeably facilitate the densification process and decrease the sintering temperature [10], open pores were apparently observed in both composites. The interface between ZrO_2_ and Si_3_N_4_ was continuous without any apparent cracks. Since the open pores are closely associated with graphene platelets, it is expected that the porosity increases with the increase in the graphene content. The porosity data, measured by both water and mercury intrusion porosimetry, are given in Table 1. A porosity close to 50% was observed for the composite with 30 wt.% MLG, which proves that MLG fillers in Si_3_N_4_-ZrO_2_ ceramics makes the densification of the composite extremely difficult. Even an addition of 5 wt.% MLG can generate a porous microstructure with about 30% porosity. Correspondingly, the densities are 2.71 g/cm^3^ and 1.84 g/cm^3^.

The morphology of the composites and the elemental distribution were investigated by TEM (Figure 3). MLG flakes distributed and embedded in the Si_3_N_4_-based matrix were clearly identified based on scanning TEM images for both composites. A certain amount of ZrO_2_ is located between the Si_3_N_4_ grains. The cross-section study of multilayered graphene flakes reveals their main presence between the rod-like Si_3_N_4_ particles. Compared with the composite with 30 wt.% MLG, more hexagonal β-Si_3_N_4_ grains were observed in the composite with 5 wt.% MLG. ZrO_2_ grains show an average size of less than 1 µm in both composites, while the size of silicon nitride rods is about 300 nm in diameter and 800 to 1200 nm in length. As shown in Figure 3, Si, N, C, Zr, Y, Al and O are the major elements detected by EDX in the TEM. Corresponding elemental mappings clearly indicate Si_3_N_4_, ZrO_2_ and MLG flakes in both 5 wt.% (Figure 3a) and 30 wt.% (Figure 3b) composites. Y distributes homogenously inside ZrO_2_, indicating the high degree of stabilization, which results in the formation of the cubic phase as proven by XRD. No transformation of ZrO_2_ is observed indicating that ZrO_2_ has no toughening effect. Al distributes around both ZrO_2_ and Si_3_N_4_; this is probably the remainder of the precipitated liquid phase. The elemental analysis reveals a low content of oxygen (2 to 4 at%) in the MLG flakes. There is no size difference observed in the TEM study for both composites. The thickness ranges mainly between 5 and 30 nm (thickness of about 20 nm (~65 carbon layers) is also confirmed by XRD, Figures S1 and S2), while the length ranges mainly between 100 and 300 nm. The quantitative size distribution of MLG flakes is summarized in Figure 4c,d. The data reveal that the carbon is mostly in the form of thin graphite.

**Figure 5:**
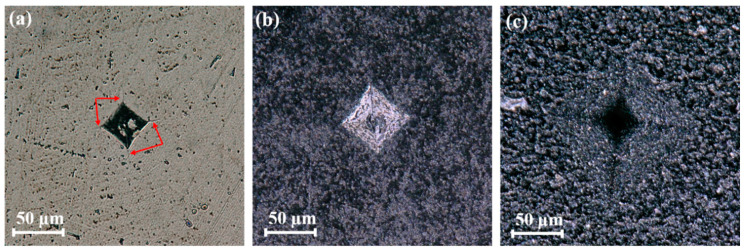
Optical microscopy images of indents after Vickers hardness measurements. (**a**) With 0 wt.% MLG, (**b**) with 5 wt.% MLG, and (**c**) with 30 wt.% MLG.

Figure 5a shows a representative optical microscopy image of a Vickers indentation site in dense Si_3_N_4_ without MLG. Classical radial cracking (extended cracks as indicated by red arrows) is clearly observed in the micrograph. Figure 5b,c show representative optical microscopy images of the Vickers indentation sites in Si_3_N_4_/MLG composites. No classical radial cracks occur in the porous composites, indicating that the Si_3_N_4_/MLG composites have potential resistance to contact or indentation damage [41]. The high porosity and shear-weak second phases (MLG) could play important roles in redistributing stress under confined shear in indentation (contact loading), resulting in the suppression of macroscopic (long) cracks. Since high porosity could deteriorate the mechanical properties of the sintered composites, potential approaches (e.g., optimizing MLG content, achieving high density, forming sandwich structure with alternating low and high MLG content layers [42,43]) can be applied to compensate the deteriorated mechanical properties.

**Figure 6:**
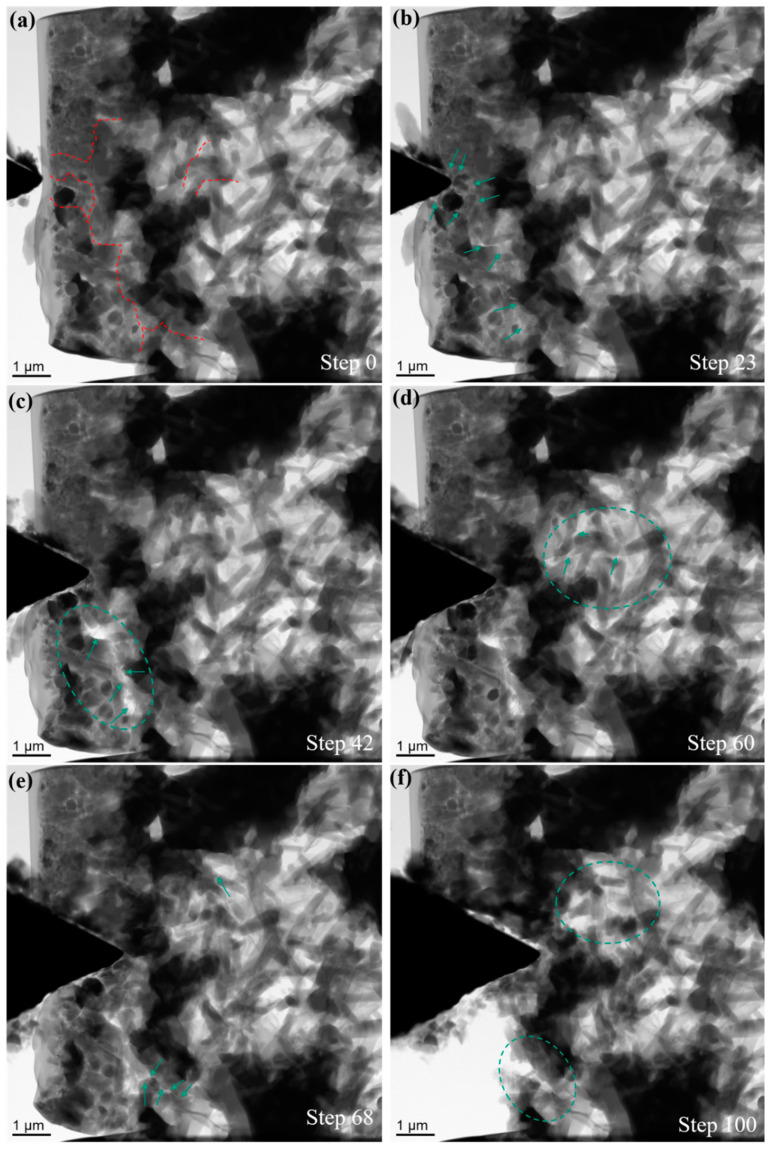
Representative TEM images selected from an in-situ experiment for a composite with 5 wt.% MLG. (**a**) Before the in-situ experiment, major cracks observed in the following experiment are highlighted by dashed lines; (**b**–**e**) selected TEM images during the in-situ experiment, crack propagation and fracture interfaces are highlighted by green arrows; (**f**) last TEM image recorded before retracting the indenter, fracture interfaces and rotated ceramic phases are highlighted by dashed ellipses.

**Figure 7:**
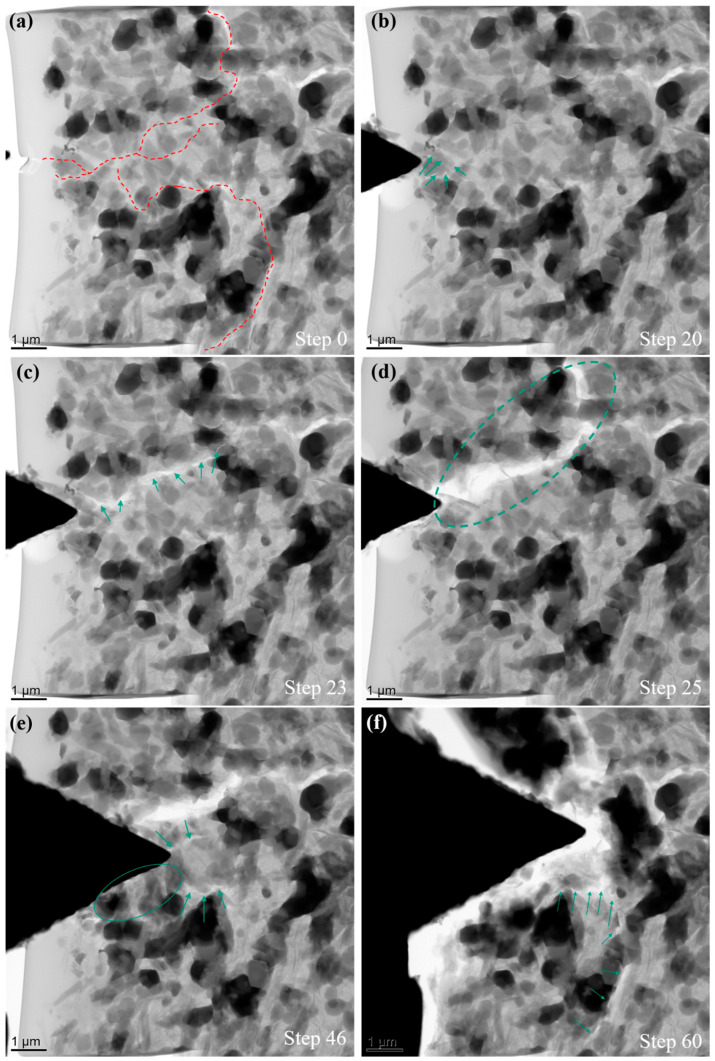
Representative TEM images selected from an in-situ experiment for a composite with 30 wt.% MLG. (**a**) Before the in-situ experiment, major cracks observed in the following experiment are highlighted by dashed lines; (**b**,**c**) the initiation and propagation of first crack, highlighted by green arrows; (**d**) delamination caused by the first long crack; (**e**,**f**) development of further cracks before retracting the indenter.

In order to gain an in-depth understanding of the mechanical behavior and the fracture mechanism of sintered composites, in-situ wedge indentation tests were performed as shown in Figure 6 and Figure 7. The corresponding videos from experiments are presented in Videos S2 and S3 (Supplementary Materials). Representative TEM images selected from an in-situ experiment for a composite with 5 wt.% MLG are shown in Figure 6. Rod-like β-Si_3_N_4_ phases with high aspect ratio account for the majority of Si_3_N_4_ phase. The positions of major cracks observed in the experiment are highlighted by dashed lines (Figure 6a). Cracks initiated in multiple locations, and propagated along weak interfaces (Figure 6b). Cracks turned towards different directions at the ceramic/MLG interface, within the ceramic phase and within the MLG platelets. Pulled out MLGs are visible in the partially fractured interface, fracture within ceramic phases is frequently observed as well (Figure 6c). The fracture surface typically looks sharp and straight in ceramic phases (Figure 6c–f). As shown in Figure 6f, a small part of the composite was completely delaminated and removed at the end of the experiment. Apart from the cracking process, shift of ceramic grains is also observed. Due to the high porosity, densification process occurs commonly in a relatively homogeneous pace. In the composite with 30 wt.% MLG, β-Si_3_N_4_ grains with low aspect ratio were mainly obtained after sintering process (Figure 7). A small amount (about 2.5%) of α-Si_3_N_4_ phase remained. The cracks propagated in a much faster pace after initiation. The cracks penetrated easily through the composite mainly within the MLG phase. A long crack within the MLG was quickly observed (step 23, Figure 7c), and subsequently a large fracture interface was formed two steps later (step 25, Figure 7d). Pulled-out MLG components are commonly visible at the fracture surface (Figure 7d). Shift of ceramic grains and a densification process were observed, but with a much faster pace. It seems that the cracks prefer to bypass the ceramic phases in the composite with 30 wt.% MLG. Such a crack propagation behavior could be caused by a high content of the weak carbon phase which forms a three-dimensional network.

The microstructure has a strong influence on the fracture behavior of the Si_3_N_4_/MLG composites. Contact loading (indentation) with highly concentrated loads act on a very small region, resulting in an intensely confined shear. Therefore, highly heterogeneous ceramic matrix composites with shear-weak graphite and high porosity here result in a considerable redistribution of stress in the region below the indentation. The behavior can be described by a distributed shear anelasticity in the form of microstructure-localized shear-sliding along numerous interfaces [41]. The high porosity (28% and 47% for 5 wt.% MLG content and 30 wt.% MLG content, respectively) improves the shear-deformability of the composites even more. This type of dispersed damage caused by significant redistributed stress consequently prevents the formation of long macro cracks (classical radial cracks), as observed in the homogenous Si_3_N_4_ ceramics (Figure 5a). Considering the fine scale of ceramic grains (about 200 nm in diameter and about 1µm in length for Si_3_N_4_, less than 1 µm in diameter for ZrO_2_) and of the carbon-based MLG as reinforcing component (tens of nm in thickness and less than 1 µm in size), it is not surprising that no macro toughening is observed, which requires this kind of carbon-based reinforcing component with larger scale (e.g., carbon fiber with a length of hundreds of micrometers [41]). The fine scale of the reinforcing component (MLG) in the composites studied here results in a small toughening zone relative to the crack size.

**Figure 8:**
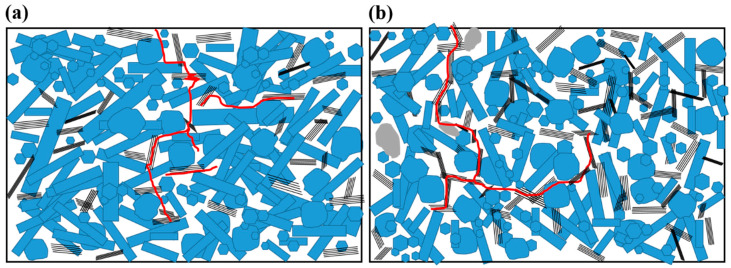
The crack propagation paths in Si_3_N_4_/MLG composites. (**a**) With 5 wt.%, (**b**) with 30 wt.%.

Although both Si_3_N_4_/MLG (5 wt.% and 30 wt.%) composites are characterized by a resistance against contact damage (Figure 5), the content of MLG plays an important role in the micro fracture behavior. In the composite with 30 wt.% MLG, the high content of the weak carbon component forms a three-dimensional network. As summarized in Table S1, a higher volume ratio (about 2:1 for Si_3_N_4_:ZrO_2_ compared to 2.65:1) of spheroid shape of ZrO_2_ with 260 nm smaller in average diameter, lower aspect ratio (5.47:1 compared to 6.77:1) of Si_3_N_4_ phase, and high porosity of about 50% facilitate the crack propagation along the three-dimensional network of the weak carbon component. The typical crack paths are sketched in Figure 8b. Since such a three-dimensional network of MLG is not formed in the composite with 5 wt.% MLG, crack deflection and crack-bridging occur locally (see Figure 8a). The cracks propagate into Si_3_N_4_ phase as well. The high porosity in the composites causes a redistribution of stress that shifts ceramic and carbon components, and consequently, leads to the direction change of cracks and new crack initiation. 

## 4. Conclusions

In summary, porous silicon nitride–zirconia–graphene composites with high graphene content (5 wt.% and 30 wt.%) were sintered by GPS. Multi-scale microscopy confirmed that all components had been dispersed evenly in the composite without obvious agglomeration. Graphene caused a reduction in the sintering additives, and therefore an increased porosity. MLG fillers in Si_3_N_4_-ZrO_2_ ceramics hampered the densification of the composites, observable by porosity values of about 30% (5 wt.% MLG) and about 50% (30 wt.% MLG). A quantitative analysis on SEM and TEM images revealed that the size distribution of the Si_3_N_4_ phase shifts towards a larger size range with increased graphene content. The higher porosity in the composite with the higher graphene content (30 wt.%) hinders the growth of the ZrO_2_ phase. The average diameters of Si_3_N_4_ grains were 221 ± 9 nm (5 wt.% MLG) and 249 ± 9 nm (30 wt.% MLG), and the average sizes of spheroid ZrO_2_ particles were 867 ± 27 nm (5 wt.% MLG) and 601 ± 19 nm (30 wt.% MLG). The volume ratio of Si_3_N_4_ and ZrO_2_ phase was about 2.65:1 in the composite with 5 wt.% MLG, and about 2:1 in the composite with 30 wt.% MLG. The MLG flakes well dispersed between ceramic grains slowed down the phase transformation from α to β-Si_3_N_4_ and the subsequent longitudinal growth of β-Si_3_N_4_ rods due to the interaction with the sintering additives, particularly visible for the composite with 30 wt.% MLG. Both Si_3_N_4_/MLG composites show a potential resistance against contact or indentation damage due to significant redistributed stress. Crack initiation and propagation, densification of the porous material and shift of ceramic components were observed in in-situ experiments. The cracks prefer to bypass ceramic components in the composite with 30 wt.% MLG, which is mainly caused by the formed three-dimensional network with a high content of weak carbon components. Without such a three-dimensional network of MLG in the composite with 5% MLG, crack deflection and crack-bridging occur locally. The cracks propagate into the Si_3_N_4_ phase as well. Redistribution of stress moves the ceramic phases and the carbon phase, which leads to the change of crack direction and to new crack initiation. Multi-scale and in-situ microscopy as a combined methodology reported in this study also provides a potential unique approach to understand the microstructure and mechanical behavior correlation for complex ceramic systems.

## Figures and Tables

**Table 1:** Density and porosity of synthesized silicon nitride–zirconia–graphene composite.

MLG Content	Density (g/cm^3^)	Porosity/Water Intrusion (%)	Porosity/Mercury Intrusion (%)
5 wt.% MLG	2.71	28	33.4
30 wt.% MLG	1.84	47	47.5

## Data Availability

The data presented in this study are available on request from the corresponding author. This data is not publicly available due to excessive size and complex format.

## Supplementary Materials

The following are available online at https://www.mdpi.com/2079-4991/11/2/285/s1, Figure S1: XRD data from the sintered silicon nitride-zirconia-graphene composite with 5 wt.% MLG, Figure S2: XRD data from the sintered silicon nitride-zirconia-graphene composite with 30 wt.% MLG, Table S1: Microstructure comparison for silicon-zirconia-graphene composites, Video S1: Tomography, Video S2: In-situ TEM for the composite with 5 wt.% MLG, Video S3: In-situ TEM for the composite with 30 wt.% MLG.

## References

[1] Hampshire S. (2007). Silicon nitride ceramics—Review of structure, processing and properties. J. Achiev. Mater. Manuf. Eng..

[2] Riley F.L. (2000). Silicon nitride and related materials. J. Am. Ceram. Soc..

[3] Ziegler G., Heinrich J., Wötting G. (1987). Relationships between processing, microstructure and properties of dense and reaction-bonded silicon nitride. J. Mater. Sci..

[4] Cygan T., Wozniak J., Kostecki M., Adamczyk-Cieslak B., Olszyna A. (2016). Influence of graphene addition and sintering temperature on physical properties of Si_3_N_4_ matrix composites. Int. J. Refract. Met. H..

[5] Klemm H. (2010). Silicon nitride for high-temperature applications. J. Am. Ceram. Soc..

[6] Ariff T.F., Shafie N.S., Zahir Z.M. (2012). Wear analysis of silicon nitride cutting tool in dry machining of T6061 aluminium alloy. Appl. Mech. Mater..

[7] Rutkowski P., Stobierski L., Zientara D., Jaworska L., Klimczyk P., Urbanik M. (2015). The influence of the graphene additive on mechanical properties and wear of hot-pressed Si_3_N_4_ matrix composites. J. Eur. Ceram. Soc..

[8] Rauta P.R., Manivasakan P., Rajendran V., Sahu B.B., Panda B.K., Mohapatra P. (2012). Phase transformation of ZrO_2_ nanoparticles produced from zircon. Phase Transit..

[9] Chevalier J., Gremillard L., Virkar A.V., Clarke D.R. (2009). The Tetragonal-Monoclinic Transformation in Zirconia: Lessons Learned and Future Trends. J. Am. Ceram. Soc..

[10] Sayyadi-Shahraki A., Rafiaei S.M., Ghadami S., Nekouee K.A. (2019). Densification and mechanical properties of spark plasma sintered Si_3_N_4_/ZrO_2_ nano-composites. J. Alloys Compd..

[11] Balázsi C., Shen Z., Kónya Z., Kasztovszky Z., Wéber F., Vértesy Z., Biró L.P., Kiricsi I., Arató P. (2005). Processing of carbon nanotube reinforced silicon nitride composites by spark plasma sintering. Compos. Sci. Technol..

[12] Ramírez C., Vega-Diaz S.M., Morelos-Gómez A., Figueiredo F.M., Terrones M., Osendi M.I., Belmonte M., Miranzo P. (2013). Synthesis of conducting graphene/Si_3_N_4_ composites by spark plasma sintering. Carbon.

[13] Balázsi K., Furkó M., Fogarassy Z., Balázsi C. (2018). Examination of milled h-BN addition on sintered Si_3_N_4_/h-BN ceramic composites. Process. Appl. Ceram..

[14] Zhu Y., Murali S., Cai W., Li X., Suk J.W., Potts J.R., Ruoff R.S. (2010). Graphene and graphene oxide: Synthesis, properties, and applications. Adv. Mater..

[15] Kvetkova L., Duszova A., Kasiarova M., Dorcakova F., Dusza J., Balazsi C. (2013). Influence of processing on fracture toughness of Si_3_N_4_+ graphene platelet composites. J. Eur. Ceram. Soc..

[16] Ramirez C., Miranzo P., Belmonte M., Osendi M.I., Poza P., Vega-Diaz S.M., Terrones M. (2014). Extraordinary toughening enhancement and flexural strength in Si_3_N_4_ composites using graphene sheets. J. Eur. Ceram. Soc..

[17] Dusza J., Morgiel J., Duszova A., Kvetkova L., Nosko M., Kun P., Balazsi C. (2012). Microstructure and fracture toughness of Si_3_N_4_+ graphene platelet composites. J. Eur. Ceram. Soc..

[18] Kvetkova L., Duszova A., Hvizdos P., Dusza J., Kun P., Balazsi C. (2012). Fracture toughness and toughening mechanisms in graphene platelet reinforced Si_3_N_4_ composites. Scripta Mater..

[19] Seiner H., Ramirez C., Koller M., Sedlak P., Landa M., Miranzo P., Belmonte M., Osendi M.I. (2015). Elastic properties of silicon nitride ceramics reinforced with graphene nanofillers. Mater. Des..

[20] Hvizdos P., Dusza J., Balazsi C. (2013). Tribological properties of Si_3_N_4_-graphene nanocomposites. J. Eur. Ceram. Soc..

[21] Balko J., Hvizdos P., Dusza J., Balazsi C., Gamcova J. (2014). Wear damage of Si_3_N_4_-graphene nanocomposites at room and elevated temperatures. J. Eur. Ceram. Soc..

[22] Rutkowski P., Stobierski L., Gorny G. (2014). Thermal stability and conductivity of hot-pressed Si_3_N_4_-graphene composites. J. Therm. Anal. Calorim..

[23] Balazsi K., Furko M., Liao Z., Gluch J., Medved D., Sedlak R., Dusza J., Zschech E., Balazsi C. (2020). Porous sandwich ceramic of layered silicon nitride-zirconia composite with various multilayered graphene content. J. Alloys Compd..

[24] Balazsi K., Furko M., Liao Z., Fogarassy Z., Medved D., Zschech E., Dusza J., Balazsi C. (2020). Graphene added multilayer ceramic sandwich (GMCS) composites: Structure, preparation and properties. J. Eur. Ceram. Soc..

[25] Cheng C., Fan R., Wang Z., Shao Q., Guo X., Xie P., Yin Y., Zhang Y., An L., Lei Y. (2017). Tunable and weakly negative permittivity in carbon/silicon nitride composites with different carbonizing temperatures. Carbon.

[26] Yang X., Li B., Zhang C., Wang S., Liu K., Zou C. (2016). Fabrication and properties of porous silicon nitride wave-transparent ceramics via gel-casting and pressureless sintering. Mater. Sci. Eng. A.

[27] Latella B., O’connor B., Padture N., Lawn B. (1997). Hertzian contact damage in porous alumina ceramics. J. Am. Ceram. Soc..

[28] She J., Yang J., Beppu Y., Ohji T. (2003). Hertzian contact damage in a highly porous silicon nitride ceramic. J. Eur. Ceram. Soc..

[29] Staub D., Meille S., Le Corre V., Rouleau L., Chevalier J. (2016). Identification of a damage criterion of a highly porous alumina ceramic. Acta Mater..

[30] Li D., Yang X., Gao S., Zheng Y. (2018). Fabrication and properties of in situ silicon nitride nanowires reinforced porous silicon nitride (SNNWs/SN) compsites. J. Eur. Ceram. Soc..

[31] Zhang J., Ye F. (2010). Effect of agarose content on microstructures and mechanical properties of porous silicon nitride ceramics produced by gelcasting. J. Zhejiang Univ. Sci. A (Appl. Phys. Eng.).

[32] Rabinskiy L., Ripetsky A., Stitnikov S., Solyaev Y., Kahramanov R. (2016). Fabrication of porous silicon nitride ceramics using binder jetting technology. IOP Conf. Ser. Mater. Sci. Eng..

[33] Balazsi C. (2012). Silicon nitride composites with different nanocarbon additives. J. Korean Ceram. Soc..

[34] Agulleiro J.I., Fernandez J.J. (2011). Fast tomographic reconstruction on multicore computers. Bioinformatics.

[35] Levin B.D.A., Jiang Y., Padgett E., Waldon S., Quammen C., Harris C., Ayachit U., Hanwell M., Ercius P., Muller D.A. (2018). Tutorial on the Visualization of Volumetric Data Using tomviz. Micros. Today.

[36] Schindelin J., Arganda-Carreras I., Frise E., Kaynig V., Longair M., Pietzsch T., Preibisch S., Rueden C., Saalfeld S., Schmid B. (2012). Fiji: An open-source platform for biological-image analysis. Nat. Methods.

[37] Awoyera P.O., Akinmusuru J.O., Dawson A.R., Ndambuki J.M., Thom N.H. (2018). Microstructural characteristics, porosity and strength development in ceramic-laterized concrete. Cem. Concr. Compos..

[38] Hnatko M., Sajgalik P., Lences Z., Salamon D., Monteverde F. (2001). Carbon reduction reaction in the Y_2_O_3_-SiO_2_ glass system at high temperature. J. Eur. Ceram. Soc..

[39] Hnatko M., Galusek D., Sajgalik P. (2004). Low-cost preparation of Si_3_N_4_-SiC micro/nano composites by in-situ carbothermal reduction of silica in silicon nitride matrix. J. Eur. Ceram. Soc..

[40] Sajgalik P., Hnatko M., Copan P., Lences Z., Huang J. (2006). Influence of graphite additives on wear properties of hot pressed Si_3_N_4_ ceramics. J. Ceram. Soc. JAPAN.

[41] Wang X., Padture N.P., Tanaka H. (2004). Contact-damage-resistant ceramic/single-wall carbon nanotubes and ceramic/graphite composites. Nat. Mater..

[42] Cheng L., Sun M., Ye F., Bai Y., Li M., Fan S., Zhang L. (2018). Structure design, fabrication, properties of laminated ceramics: A review. Int. J. Light. Mater. Manuf..

[43] Sun M., Bai Y., Li M., Fan S., Cheng L. (2018). Structural design and energy absorption mechanism of laminated SiC/BN ceramics. J. Eur. Ceram. Soc..

