# Supplementary material for: Microstructure and Fracture Mechanism Investigation of Porous Silicon Nitride–Zirconia–Graphene Composite Using Multi-Scale and In-Situ Microscopy
Source: Nanomaterials (Basel). 2021 Jan 22;11(2):285. doi: 10.3390/nano11020285 (PMC7911286; doi:10.3390/nano11020285)
Supplement: Supplementary file 1 [file nanomaterials-11-00285-s001.zip › Supplementary files_after proofreading/Supplementary material_after proofreading.docx]

**Supplementary material**

Microstructure and Fracture Mechanism Investigation of Porous Silicon Nitride–Zirconia–Graphene Composite Using Multi-Scale and In-Situ Microscopy

**Zhongquan Liao ^1,^*, Yvonne Standke ^1^, Jürgen Gluch ^1^, Katalin Balázsi ^2^, Onkar Pathak ^1^, Sören Höhn ^3^, Mathias Herrmann ^3^, Stephan Werner ^4^, Ján Dusza ^5^, Csaba Balázsi ^2^ and Ehrenfried Zschech ^1^**

^1^ Fraunhofer Institute for Ceramic Technologies and Systems IKTS, Maria-Reiche-Straße 2, 01109 Dresden, Germany; yvonne.standke@de.bosch.com (Y.S.); juergen.gluch@ikts.fraunhofer.de (J.G.); onkar_pathak@ymail.com (O.P.); ehrenfried.zschech@ikts.fraunhofer.de (E.Z.)

^2^ Centre for Energy Research, Konkoly-Thege str. 29-33, 1121 Budapest, Hungary; balazsi.katalin@ek-cer.hu (K.B.); balazsi.csaba@ek-cer.hu (C.B.)

^3^ Fraunhofer Institute for Ceramic Technologies and Systems IKTS, Winterbergstraße 28, 01277 Dresden, Germany; soeren.hoehn@ikts.fraunhofer.de (S.H.); mathias.herrmann@ikts.fraunhofer.de (M.H.)

^4^ Helmholtz Zentrum Berlin, Albert-Einstein-Straße 15, 12489 Berlin, Germany; stephan.werner@helmholtz-berlin.de (S.W.)

^5^ Institute of Materials Research, Slovak Academy of Sciences, Watsonova 47, 040 01 Košice, Slovakia; duszaj@yahoo.com (J.D.)

***** Correspondence: zhongquan.liao@ikts.fraunhofer.de; Tel.: +49-(0)351-888-15598 (Z.L.)

**Figure S1.** XRD data from the sintered silicon nitride-zirconia-graphene composite with 5 wt.% MLG. The data was acquired from the surface of sintered composite.


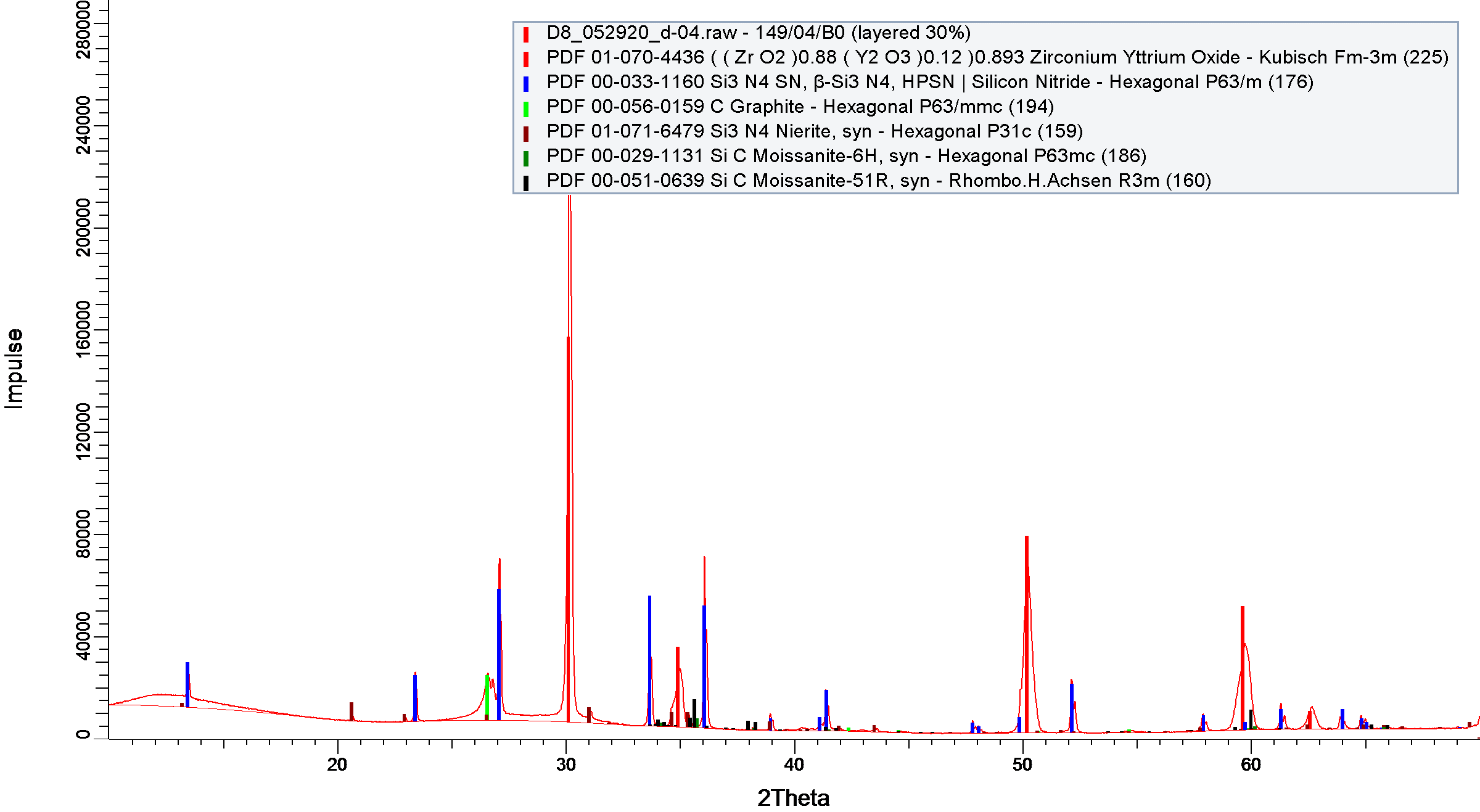


**Figure S2.** XRD data from the sintered silicon nitride-zirconia-graphene composite with 30 wt.% MLG. The data was acquired from the surface of sintered composite.

**Table S1.** Microstructure comparison for silicon-zirconia-graphene composites.

| **MLG content** | **Volume ratio (Si_3_N_4_/ZrO_2_)** | **Size of spheroid ZrO_2_ (nm)** | **Aspect ratio of Si_3_N_4_ phase** | **Porosity** |
| --- | --- | --- | --- | --- |
| 5 wt.% MLG | 2.65:1 | 867±27 | 6.77:1 | ~ 30 % |
| 30 wt.% MLG | 2:1 | 601±19 | 5.47:1 | ~ 50 % |
